# 2-[1-(4-Ethoxy­phen­yl)-2-oxo-4-styryl­azetidin-3-yl]isoindoline-1,3-dione

**DOI:** 10.1107/S1600536808011586

**Published:** 2008-04-26

**Authors:** Mehmet Akkurt, Selvi Karaca, Ali Asghar Jarrahpour, Maaroof Zarei, Orhan Büyükgüngör

**Affiliations:** a Department of Physics, Faculty of Arts and Sciences, Erciyes University, 38039 Kayseri, Turkey; b Department of Chemistry, College of Sciences, Shiraz University, 71454 Shiraz, Iran; c Department of Physics, Faculty of Arts and Sciences, Ondokuz Mayıs University, 55139 Samsun, Turkey

## Abstract

The title compound, C_27_H_22_N_2_O_4_, contains a nearly planar four-membered β-lactam ring, which makes dihedral angles of 74.64 (12), 1.70 (11) and 73.67 (12)° with the nine-membered ring system, the benzene ring and the phenyl ring, respectively. The crystal structure is stabilized by C—H⋯O and C—H⋯π inter­actions and a π–π inter­action [centroid–centroid distance = 3.4505 (19) Å] is also present.

## Related literature

For related structures, see: Pınar *et al.* (2006 ▶); Akkurt *et al.* (2007 ▶). For background, see: Halve *et al.* (2007 ▶); Aoyama *et al.* (2001 ▶). For related literature, see: Jarrahpour & Zarei (2007 ▶).
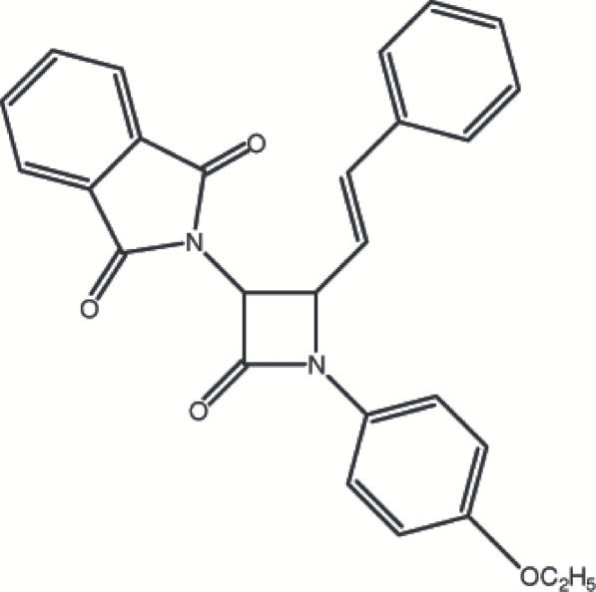


## Experimental

### 

#### Crystal data

C_27_H_22_N_2_O_4_
*M* *_r_* = 438.47
Monoclinic, 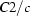

*a* = 33.7560 (17) Å
*b* = 7.0403 (2) Å
*c* = 31.0482 (17) Å
β = 140.454 (3)°
*V* = 4698.0 (5) Å^3^
*Z* = 8
Mo *K*α radiation
μ = 0.08 mm^−1^
*T* = 293 (2) K
0.53 × 0.45 × 0.14 mm

#### Data collection

Stoe IPDS-2 diffractometer
Absorption correction: none
22562 measured reflections
4934 independent reflections
3071 reflections with *I* > 2σ(*I*)
*R*_int_ = 0.052

#### Refinement

*R*[*F* ^2^ > 2σ(*F* ^2^)] = 0.042
*wR*(*F* ^2^) = 0.099
*S* = 0.96
4934 reflections
299 parameters
H-atom parameters constrained
Δρ_max_ = 0.17 e Å^−3^
Δρ_min_ = −0.11 e Å^−3^

### 

Data collection: *X-AREA* (Stoe & Cie, 2002 ▶); cell refinement: *X-AREA*; data reduction: *X-RED32* (Stoe & Cie, 2002 ▶); program(s) used to solve structure: *SIR97* (Altomare *et al.*, 1999 ▶); program(s) used to refine structure: *SHELXL97* (Sheldrick, 2008 ▶); molecular graphics: *ORTEP-3* (Farrugia, 1997 ▶); software used to prepare material for publication: *WinGX* (Farrugia, 1999 ▶).

## Supplementary Material

Crystal structure: contains datablocks global, I. DOI: 10.1107/S1600536808011586/hb2715sup1.cif

Structure factors: contains datablocks I. DOI: 10.1107/S1600536808011586/hb2715Isup2.hkl

Additional supplementary materials:  crystallographic information; 3D view; checkCIF report

## Figures and Tables

**Table 1:** Hydrogen-bond geometry (Å, °)

*D*—H⋯*A*	*D*—H	H⋯*A*	*D*⋯*A*	*D*—H⋯*A*
C3—H3⋯O1^i^	0.93	2.53	3.283 (3)	138
C5—H5⋯O3^ii^	0.93	2.47	3.257 (3)	142
C13—H13⋯O3	0.93	2.53	3.139 (2)	123
C20—H20⋯O2^iii^	0.93	2.51	3.374 (2)	156
C9—H9⋯*Cg*1^iv^	0.98	2.84	3.7938 (16)	166
C19—H19*C*⋯*Cg*2^v^	0.96	2.82	3.633 (4)	143

Symmetry codes: (i) 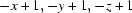; (ii) 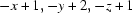; (iii) 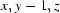; (iv) 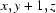; (v) 
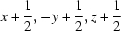
. Cg1 is the centroid of atoms C12–C17 and Cg2 is the centroid of atoms C2–C7.

**Fig. 1.**
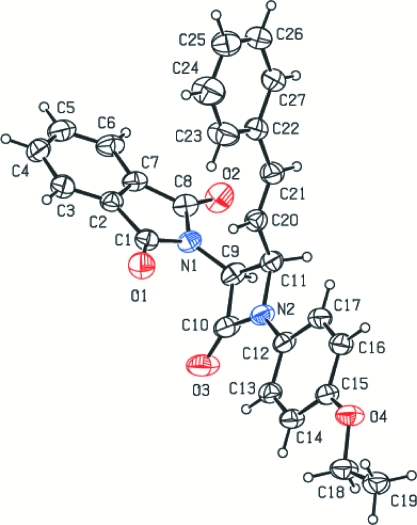
View of the molecular structure of (I), with 30% probability displacement ellipsoids for the non-hydrogen atoms.

**Fig. 2.**
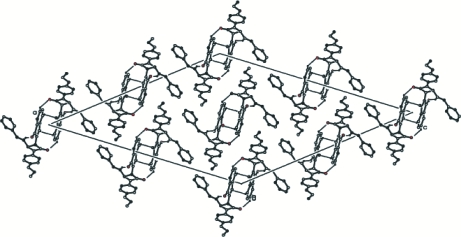
View of the packing and hydrogen bonding interactions for (I). H atoms not involved in hydrogen bonding have been omitted for clarity.

## supplementary crystallographic information

### Comment

The 2-azetidinone ring system is the common structural feature of a number of broad spectrum β-lactam antibiotics (Halve *et al.*, 2007) and also possesses other pharmalogical properties (Aoyama *et al.*, 2001).  As part of our onging studies of such materials (Pınar *et al.*, 2006; Akkurt *et al.*, 2007), we now report the synthesis and structure of the title compound, (I), (Fig. 1).

The four-membered β-lactam ring in (I) is nearly planar, with maximum deviations of 0.022 (1) Å for N2 and -0.021 (2) Å for C10. Within the lactam ring, the bond lengths are similar to those observed in our previous studies (Pınar *et al.*, 2006; Akkurt *et al.*, 2007).

The four-membered β-lactam ring (N2/C9–C11) in (I) makes dihedral angles of 74.64 (12), 1.70 (11) and 73.67 (12)° with the nine-membered ring system (A: N1/C1–C8) {max. deviations from planarity = 0.048 (2) for N1 and -0.029 (3) Å for C2 and C7}, the benzene ring (B: C12–C17) and the phenyl ring (C: C22–C27), respectively. The other dihedral angles are A/B = 74.74 (9), A/C = 55.97 (11) and B/C = 74.84 (10)°. The sum of the bond angles about atom N2 is 360.0°.

The packing and hydrogen bonding of the title compound is shown in Fig. 2. The crystal structure is stabilized by inter- and intramolecular C—H···O interactions and C—H···π contacts (Table 1). Finally, an aromatic π—π stacking interaction {*C_g_*3···*C_g_*2(1 – *x*, 2 – *y*,1 – *z*) = 3.4505 (19)Å, where *C_g_3 is* the centroid of the N1/C1/C2/C7/C8 five-membered ring} is observed in the crystal structure.

### Experimental

A solution of Schiff base (4-cinnamylidene)-(4-ethoxyphenyl)amine (1.0 eq.) was stirred with Phthaloylglycine (1.5 eq.), p-toluenesulfonyl chloride (1.5 eq.) and triethylamine (5 eq.) in dry CH_2_Cl_2_ at room temperature. After 10 h, the mixture was washed with saturated sodium bicarbonate solution and brine, dried over sodium sulfate and the solvent was evaporated to give the crude product which was then purified by recrystalization from EtOAc (Jarrahpour & Zarei, 2007) [mp: 434 -436 K]. IR (CHCl_3_) cm^-1^: 1724.2, 1758.5 (CO, phth), 1774.7 (CO, β-lactam); ^1^H NMR (250 MHz, CDCl_3_) δ 1.37 (Me, t, 3H), 2.33 (Me, s, 3H), 3.97 (OCH_2_, q, 2H), 5.03 (H-4, dd, 1H, J=5.5, 8.5), 5.68 (H-3, d, 1H, J=5.5), 6.32 (H-5, dd, J=8.5, 16.0), 6.85 (H-6, d, 1H, J=9.0), 7.19-7.82 (ArH, m, 13H); ^13^C NMR (62.9 MHz, CDCl_3_) δ 14.78 (Me), 57.69 (OCH_2_), 61.04 (C-4), 63.67 (C-3), 114.99-155.82 (C=C, aromatic carbons), 160.56 (CO, phth), 167.28 (CO, β-lactam); GC-MS m/z = 438 [M^+^]. Analysis calculated for C_27_H_22_N_2_O_4_: C 73.96, H 5.06, N 6.39%. Found: C 74.02, H 5.09, N 6.33%.

### Refinement

All the H atoms were geometrically generated (C–H = 0.93–0.98 Å) and refined as riding with *U*_iso_(H) = 1.2*U*_eq_(C) or 1.5*U*_eq_(methyl C).

### Crystal data

**Table:**

C_27_H_22_N_2_O_4_	*F*_000_ = 1840
*M**_r_* = 438.47	*D*_x_ = 1.240 Mg m^−^^3^
Monoclinic, *C*2/*c*	Mo *K*α radiation λ = 0.71073 Å
Hall symbol: -C 2yc	Cell parameters from 19454 reflections
*a* = 33.7560 (17) Å	θ = 1.3–27.3º
*b* = 7.0403 (2) Å	µ = 0.08 mm^−^^1^
*c* = 31.0482 (17) Å	*T* = 293 (2) K
β = 140.454 (3)º	Prism, colourless
*V* = 4698.0 (5) Å^3^	0.53 × 0.45 × 0.14 mm
*Z* = 8	

### Data collection

**Table:**

Stoe IPDS-2 diffractometer	3071 reflections with *I* > 2σ(*I*)
Monochromator: plane graphite	*R*_int_ = 0.052
Detector resolution: 6.67 pixels mm^-1^	θ_max_ = 26.8º
*T* = 293(2) K	θ_min_ = 1.4º
ω scans	*h* = −42→42
Absorption correction: none	*k* = −8→8
22562 measured reflections	*l* = −39→39
4934 independent reflections	

### Refinement

**Table:**

Refinement on *F*^2^	Hydrogen site location: inferred from neighbouring sites
Least-squares matrix: full	H-atom parameters constrained
*R*[*F*^2^ > 2σ(*F*^2^)] = 0.042	*w* = 1/[σ^2^(*F*_o_^2^) + (0.05*P*)^2^] where *P* = (*F*_o_^2^ + 2*F*_c_^2^)/3
*wR*(*F*^2^) = 0.099	(Δ/σ)_max_ < 0.001
*S* = 0.97	Δρ_max_ = 0.17 e Å^−^^3^
4934 reflections	Δρ_min_ = −0.11 e Å^−^^3^
299 parameters	Extinction correction: SHELXL97 (Sheldrick, 2008), Fc*=kFc[1+0.001xFc^2^λ^3^/sin(2θ)]^-1/4^
Primary atom site location: structure-invariant direct methods	Extinction coefficient: 0.0032 (2)
Secondary atom site location: difference Fourier map	

### Special details

**Table:**

Geometry. Bond distances, angles *etc*. have been calculated using the rounded fractional coordinates. All su's are estimated from the variances of the (full) variance-covariance matrix. The cell e.s.d.'s are taken into account in the estimation of distances, angles and torsion angles
Refinement. Refinement on *F*^2^ for ALL reflections except those flagged by the user for potential systematic errors. Weighted *R*-factors *wR* and all goodnesses of fit *S* are based on *F*^2^, conventional *R*-factors *R* are based on *F*, with *F* set to zero for negative *F*^2^. The observed criterion of *F*^2^ > σ(*F*^2^) is used only for calculating -*R*-factor-obs *etc*. and is not relevant to the choice of reflections for refinement. *R*-factors based on *F*^2^ are statistically about twice as large as those based on *F*, and *R*-factors based on ALL data will be even larger.

### Fractional atomic coordinates and isotropic or equivalent isotropic displacement parameters (Å^2^)

**Table:**

	*x*	*y*	*z*	*U*_iso_*/*U*_eq_	
O1	0.52753 (6)	0.55052 (15)	0.57556 (7)	0.0765 (5)	
O2	0.54065 (6)	1.14698 (16)	0.64241 (7)	0.0887 (5)	
O3	0.67821 (7)	0.6142 (2)	0.70712 (8)	0.1034 (6)	
O4	0.74347 (5)	−0.13874 (15)	0.89253 (6)	0.0729 (4)	
N1	0.54930 (5)	0.83949 (16)	0.62525 (6)	0.0555 (4)	
N2	0.64313 (6)	0.53412 (18)	0.74672 (7)	0.0617 (4)	
C1	0.51510 (7)	0.7165 (2)	0.57070 (8)	0.0562 (5)	
C2	0.46331 (7)	0.8305 (2)	0.50943 (8)	0.0554 (5)	
C3	0.41750 (8)	0.7826 (3)	0.44180 (9)	0.0708 (6)	
C4	0.37641 (8)	0.9267 (3)	0.39554 (10)	0.0814 (8)	
C5	0.38151 (9)	1.1085 (3)	0.41602 (11)	0.0795 (8)	
C6	0.42714 (8)	1.1549 (2)	0.48327 (10)	0.0714 (7)	
C7	0.46791 (7)	1.0133 (2)	0.52986 (8)	0.0566 (5)	
C8	0.52172 (7)	1.0197 (2)	0.60414 (9)	0.0598 (5)	
C9	0.60433 (7)	0.7905 (2)	0.69508 (8)	0.0641 (5)	
C10	0.64799 (8)	0.6362 (3)	0.71402 (9)	0.0719 (6)	
C11	0.59771 (7)	0.6599 (2)	0.72999 (8)	0.0597 (5)	
C12	0.67075 (7)	0.3647 (2)	0.78458 (8)	0.0567 (5)	
C13	0.71296 (7)	0.2662 (2)	0.79469 (8)	0.0597 (5)	
C14	0.73897 (7)	0.0980 (2)	0.83145 (8)	0.0601 (5)	
C15	0.72250 (7)	0.0284 (2)	0.85749 (8)	0.0580 (5)	
C16	0.68038 (7)	0.1281 (2)	0.84731 (9)	0.0639 (6)	
C17	0.65484 (7)	0.2938 (2)	0.81140 (8)	0.0640 (6)	
C18	0.79389 (8)	−0.2335 (3)	0.91444 (10)	0.0753 (7)	
C19	0.81168 (9)	−0.3978 (3)	0.95687 (10)	0.0799 (7)	
C20	0.53523 (7)	0.5771 (2)	0.68565 (8)	0.0564 (5)	
C21	0.49520 (7)	0.6482 (2)	0.67920 (9)	0.0672 (6)	
C22	0.43110 (8)	0.5844 (2)	0.63340 (9)	0.0655 (6)	
C23	0.40642 (9)	0.4242 (3)	0.59366 (12)	0.0936 (8)	
C24	0.34510 (11)	0.3722 (3)	0.54989 (13)	0.1084 (10)	
C25	0.30869 (9)	0.4779 (3)	0.54607 (12)	0.0919 (8)	
C26	0.33206 (9)	0.6339 (3)	0.58488 (10)	0.0815 (7)	
C27	0.39250 (8)	0.6879 (3)	0.62798 (9)	0.0726 (6)	
H3	0.41420	0.66050	0.42780	0.0850*	
H4	0.34460	0.89950	0.34950	0.0980*	
H5	0.35350	1.20120	0.38360	0.0950*	
H6	0.43060	1.27740	0.49710	0.0860*	
H9	0.62800	0.90510	0.72220	0.0770*	
H11	0.61500	0.71940	0.77040	0.0720*	
H13	0.72390	0.31270	0.77680	0.0720*	
H14	0.76750	0.03250	0.83850	0.0720*	
H16	0.66940	0.08180	0.86510	0.0770*	
H17	0.62660	0.35940	0.80490	0.0770*	
H18A	0.82890	−0.14760	0.94060	0.0900*	
H18B	0.78150	−0.27720	0.87570	0.0900*	
H19A	0.77670	−0.48160	0.93060	0.0960*	
H19B	0.82430	−0.35290	0.99530	0.0960*	
H19C	0.84560	−0.46500	0.97210	0.0960*	
H20	0.52400	0.46840	0.66110	0.0680*	
H21	0.50880	0.75120	0.70680	0.0810*	
H23	0.43100	0.35050	0.59620	0.1120*	
H24	0.32880	0.26450	0.52300	0.1300*	
H25	0.26760	0.44230	0.51660	0.1100*	
H26	0.30720	0.70510	0.58250	0.0980*	
H27	0.40790	0.79670	0.65410	0.0870*	

### Atomic displacement parameters (Å^2^)

**Table:**

	*U*^11^	*U*^22^	*U*^33^	*U*^12^	*U*^13^	*U*^23^
O1	0.0929 (9)	0.0524 (6)	0.0847 (9)	0.0128 (6)	0.0686 (8)	0.0056 (6)
O2	0.0932 (9)	0.0554 (6)	0.0838 (9)	0.0000 (6)	0.0594 (8)	−0.0081 (7)
O3	0.0824 (9)	0.1325 (11)	0.1212 (12)	0.0437 (8)	0.0853 (10)	0.0642 (9)
O4	0.0679 (7)	0.0707 (7)	0.0925 (9)	0.0208 (6)	0.0651 (7)	0.0242 (6)
N1	0.0501 (7)	0.0471 (6)	0.0569 (8)	0.0026 (5)	0.0380 (7)	0.0046 (6)
N2	0.0499 (7)	0.0691 (8)	0.0602 (8)	0.0122 (6)	0.0409 (7)	0.0166 (7)
C1	0.0574 (9)	0.0528 (8)	0.0646 (10)	0.0019 (7)	0.0487 (9)	0.0029 (8)
C2	0.0488 (8)	0.0622 (9)	0.0601 (10)	0.0012 (7)	0.0433 (8)	0.0039 (8)
C3	0.0620 (10)	0.0890 (11)	0.0675 (12)	−0.0043 (9)	0.0515 (10)	−0.0026 (10)
C4	0.0527 (10)	0.1283 (17)	0.0578 (11)	0.0072 (11)	0.0412 (10)	0.0174 (11)
C5	0.0597 (11)	0.0968 (14)	0.0827 (15)	0.0211 (10)	0.0551 (12)	0.0319 (11)
C6	0.0641 (10)	0.0658 (10)	0.0866 (14)	0.0161 (8)	0.0587 (11)	0.0234 (9)
C7	0.0491 (8)	0.0547 (8)	0.0671 (10)	0.0053 (7)	0.0451 (9)	0.0103 (7)
C8	0.0593 (9)	0.0477 (8)	0.0698 (11)	−0.0010 (7)	0.0491 (10)	0.0023 (8)
C9	0.0488 (8)	0.0616 (9)	0.0589 (10)	−0.0025 (7)	0.0355 (8)	0.0039 (8)
C10	0.0504 (9)	0.0870 (12)	0.0687 (11)	0.0113 (8)	0.0434 (9)	0.0225 (9)
C11	0.0522 (8)	0.0608 (8)	0.0548 (9)	0.0044 (7)	0.0383 (8)	0.0025 (7)
C12	0.0421 (7)	0.0642 (9)	0.0496 (9)	0.0026 (7)	0.0316 (7)	0.0061 (7)
C13	0.0462 (8)	0.0729 (9)	0.0568 (9)	0.0006 (7)	0.0389 (8)	0.0050 (8)
C14	0.0456 (8)	0.0676 (9)	0.0649 (10)	0.0045 (7)	0.0420 (8)	0.0050 (8)
C15	0.0477 (8)	0.0627 (9)	0.0574 (10)	0.0050 (7)	0.0389 (8)	0.0057 (7)
C16	0.0607 (9)	0.0717 (10)	0.0670 (11)	0.0111 (8)	0.0513 (9)	0.0141 (8)
C17	0.0562 (9)	0.0744 (10)	0.0639 (10)	0.0152 (8)	0.0470 (9)	0.0130 (8)
C18	0.0659 (10)	0.0775 (11)	0.0890 (13)	0.0206 (9)	0.0614 (11)	0.0174 (10)
C19	0.0763 (12)	0.0824 (11)	0.0857 (13)	0.0266 (9)	0.0637 (12)	0.0206 (10)
C20	0.0555 (9)	0.0539 (8)	0.0560 (9)	0.0039 (7)	0.0420 (8)	0.0030 (7)
C21	0.0574 (9)	0.0721 (10)	0.0643 (11)	−0.0014 (8)	0.0449 (9)	−0.0129 (8)
C22	0.0579 (9)	0.0755 (10)	0.0638 (10)	−0.0002 (8)	0.0471 (9)	−0.0060 (8)
C23	0.0762 (13)	0.0912 (13)	0.1146 (17)	−0.0156 (10)	0.0739 (14)	−0.0352 (12)
C24	0.0876 (15)	0.1084 (16)	0.124 (2)	−0.0347 (13)	0.0802 (16)	−0.0474 (14)
C25	0.0647 (11)	0.1170 (16)	0.0931 (15)	−0.0159 (12)	0.0606 (12)	−0.0111 (13)
C26	0.0662 (11)	0.1067 (14)	0.0831 (13)	0.0048 (11)	0.0606 (11)	0.0017 (12)
C27	0.0659 (11)	0.0863 (11)	0.0715 (11)	0.0024 (9)	0.0545 (10)	−0.0063 (9)

### Geometric parameters (Å, °)

**Table:**

O1—C1	1.211 (2)	C21—C22	1.470 (4)
O2—C8	1.205 (2)	C22—C23	1.380 (3)
O3—C10	1.203 (5)	C22—C27	1.385 (4)
O4—C15	1.3717 (18)	C23—C24	1.388 (5)
O4—C18	1.421 (4)	C24—C25	1.362 (6)
N1—C1	1.3913 (19)	C25—C26	1.347 (3)
N1—C8	1.402 (2)	C26—C27	1.374 (4)
N1—C9	1.435 (2)	C3—H3	0.9300
N2—C10	1.353 (3)	C4—H4	0.9300
N2—C11	1.475 (3)	C5—H5	0.9300
N2—C12	1.408 (2)	C6—H6	0.9300
C1—C2	1.480 (2)	C9—H9	0.9800
C2—C3	1.381 (2)	C11—H11	0.9800
C2—C7	1.390 (2)	C13—H13	0.9300
C3—C4	1.394 (3)	C14—H14	0.9300
C4—C5	1.380 (3)	C16—H16	0.9300
C5—C6	1.371 (3)	C17—H17	0.9300
C6—C7	1.382 (2)	C18—H18A	0.9700
C7—C8	1.470 (2)	C18—H18B	0.9700
C9—C10	1.537 (4)	C19—H19A	0.9600
C9—C11	1.563 (3)	C19—H19B	0.9600
C11—C20	1.485 (3)	C19—H19C	0.9600
C12—C13	1.386 (4)	C20—H20	0.9300
C12—C17	1.386 (4)	C21—H21	0.9300
C13—C14	1.389 (2)	C23—H23	0.9300
C14—C15	1.377 (4)	C24—H24	0.9300
C15—C16	1.386 (4)	C25—H25	0.9300
C16—C17	1.366 (2)	C26—H26	0.9300
C18—C19	1.488 (3)	C27—H27	0.9300
C20—C21	1.304 (4)		
			
C15—O4—C18	118.5 (2)	C24—C25—C26	120.1 (3)
C1—N1—C8	111.45 (13)	C25—C26—C27	120.2 (3)
C1—N1—C9	125.59 (13)	C22—C27—C26	121.6 (2)
C8—N1—C9	122.91 (13)	C2—C3—H3	122.00
C10—N2—C11	96.40 (17)	C4—C3—H3	122.00
C10—N2—C12	133.7 (3)	C3—C4—H4	119.00
C11—N2—C12	129.9 (2)	C5—C4—H4	119.00
O1—C1—N1	124.49 (15)	C4—C5—H5	119.00
O1—C1—C2	129.48 (15)	C6—C5—H5	119.00
N1—C1—C2	106.03 (13)	C5—C6—H6	121.00
C1—C2—C3	130.54 (16)	C7—C6—H6	121.00
C1—C2—C7	108.06 (14)	N1—C9—H9	110.00
C3—C2—C7	121.35 (16)	C10—C9—H9	110.00
C2—C3—C4	116.54 (19)	C11—C9—H9	110.00
C3—C4—C5	121.93 (19)	N2—C11—H11	112.00
C4—C5—C6	121.16 (19)	C9—C11—H11	112.00
C5—C6—C7	117.70 (16)	C20—C11—H11	112.00
C2—C7—C6	121.31 (15)	C12—C13—H13	120.00
C2—C7—C8	108.22 (14)	C14—C13—H13	120.00
C6—C7—C8	130.44 (15)	C13—C14—H14	120.00
O2—C8—N1	123.78 (16)	C15—C14—H14	120.00
O2—C8—C7	130.10 (16)	C15—C16—H16	120.00
N1—C8—C7	106.12 (13)	C17—C16—H16	120.00
N1—C9—C10	119.36 (17)	C12—C17—H17	120.00
N1—C9—C11	118.6 (2)	C16—C17—H17	120.00
C10—C9—C11	85.79 (16)	O4—C18—H18A	110.00
O3—C10—N2	132.5 (2)	O4—C18—H18B	110.00
O3—C10—C9	135.9 (2)	C19—C18—H18A	110.00
N2—C10—C9	91.6 (2)	C19—C18—H18B	110.00
N2—C11—C9	86.09 (19)	H18A—C18—H18B	109.00
N2—C11—C20	115.99 (14)	C18—C19—H19A	109.00
C9—C11—C20	116.63 (14)	C18—C19—H19B	110.00
N2—C12—C13	121.1 (2)	C18—C19—H19C	109.00
N2—C12—C17	119.5 (2)	H19A—C19—H19B	110.00
C13—C12—C17	119.40 (16)	H19A—C19—H19C	109.00
C12—C13—C14	120.1 (2)	H19B—C19—H19C	109.00
C13—C14—C15	120.0 (2)	C11—C20—H20	118.00
O4—C15—C14	125.2 (2)	C21—C20—H20	118.00
O4—C15—C16	115.3 (2)	C20—C21—H21	116.00
C14—C15—C16	119.57 (16)	C22—C21—H21	116.00
C15—C16—C17	120.6 (3)	C22—C23—H23	120.00
C12—C17—C16	120.3 (3)	C24—C23—H23	120.00
O4—C18—C19	107.8 (3)	C23—C24—H24	120.00
C11—C20—C21	123.68 (16)	C25—C24—H24	120.00
C20—C21—C22	127.56 (17)	C24—C25—H25	120.00
C21—C22—C23	122.6 (3)	C26—C25—H25	120.00
C21—C22—C27	120.12 (16)	C25—C26—H26	120.00
C23—C22—C27	117.3 (3)	C27—C26—H26	120.00
C22—C23—C24	120.5 (3)	C22—C27—H27	119.00
C23—C24—C25	120.4 (2)	C26—C27—H27	119.00
			
C18—O4—C15—C16	−170.02 (15)	C4—C5—C6—C7	0.1 (5)
C18—O4—C15—C14	11.6 (2)	C5—C6—C7—C8	178.3 (3)
C15—O4—C18—C19	174.18 (14)	C5—C6—C7—C2	0.6 (5)
C9—N1—C1—C2	−179.5 (2)	C6—C7—C8—N1	−175.1 (3)
C9—N1—C1—O1	0.5 (5)	C2—C7—C8—N1	2.8 (3)
C1—N1—C9—C10	30.8 (4)	C6—C7—C8—O2	5.8 (6)
C8—N1—C9—C10	−152.3 (2)	C2—C7—C8—O2	−176.3 (3)
C8—N1—C1—C2	3.2 (3)	C10—C9—C11—C20	−114.35 (19)
C1—N1—C8—O2	175.4 (3)	N1—C9—C10—O3	60.1 (3)
C9—N1—C8—O2	−1.9 (5)	N1—C9—C11—C20	7.0 (3)
C1—N1—C8—C7	−3.8 (3)	C11—C9—C10—O3	−179.3 (2)
C9—N1—C8—C7	178.9 (2)	N1—C9—C11—N2	124.16 (17)
C8—N1—C1—O1	−176.8 (3)	C10—C9—C11—N2	2.85 (12)
C8—N1—C9—C11	105.6 (2)	N1—C9—C10—N2	−123.7 (2)
C1—N1—C9—C11	−71.3 (3)	C11—C9—C10—N2	−3.10 (13)
C10—N2—C12—C17	−179.02 (18)	C9—C11—C20—C21	−98.1 (3)
C11—N2—C12—C17	0.9 (3)	N2—C11—C20—C21	162.74 (18)
C12—N2—C11—C9	176.79 (16)	C13—C12—C17—C16	−0.2 (2)
C10—N2—C12—C13	0.1 (3)	N2—C12—C13—C14	−179.13 (15)
C11—N2—C12—C13	−179.98 (15)	N2—C12—C17—C16	178.93 (15)
C12—N2—C10—O3	−0.4 (4)	C17—C12—C13—C14	0.0 (2)
C12—N2—C11—C20	−65.4 (2)	C12—C13—C14—C15	0.4 (2)
C10—N2—C11—C20	114.56 (19)	C13—C14—C15—C16	−0.6 (2)
C10—N2—C11—C9	−3.25 (13)	C13—C14—C15—O4	177.73 (15)
C11—N2—C10—O3	179.7 (2)	C14—C15—C16—C17	0.4 (3)
C11—N2—C10—C9	3.30 (14)	O4—C15—C16—C17	−178.09 (15)
C12—N2—C10—C9	−176.74 (17)	C15—C16—C17—C12	0.0 (3)
N1—C1—C2—C7	−1.4 (3)	C11—C20—C21—C22	175.49 (18)
N1—C1—C2—C3	176.2 (3)	C20—C21—C22—C23	5.4 (3)
O1—C1—C2—C7	178.6 (3)	C20—C21—C22—C27	−173.5 (2)
O1—C1—C2—C3	−3.8 (6)	C21—C22—C23—C24	−178.4 (2)
C3—C2—C7—C6	−0.6 (5)	C27—C22—C23—C24	0.6 (4)
C1—C2—C3—C4	−177.3 (3)	C21—C22—C27—C26	179.0 (2)
C7—C2—C3—C4	−0.1 (5)	C23—C22—C27—C26	0.0 (3)
C3—C2—C7—C8	−178.7 (3)	C22—C23—C24—C25	−0.6 (4)
C1—C2—C7—C8	−0.9 (3)	C23—C24—C25—C26	0.0 (4)
C1—C2—C7—C6	177.2 (3)	C24—C25—C26—C27	0.6 (4)
C2—C3—C4—C5	0.7 (5)	C25—C26—C27—C22	−0.6 (4)
C3—C4—C5—C6	−0.7 (6)		

### Hydrogen-bond geometry (Å, °)

**Table:**

*D*—H···*A*	*D*—H	H···*A*	*D*···*A*	*D*—H···*A*
C3—H3···O1^i^	0.93	2.53	3.283 (3)	138
C5—H5···O3^ii^	0.93	2.47	3.257 (3)	142
C13—H13···O3	0.93	2.53	3.139 (2)	123
C20—H20···O2^iii^	0.93	2.51	3.374 (2)	156
C9—H9···C_g_1^iv^	0.98	2.84	3.7938 (16)	166
C19—H19C···C_g_2^v^	0.96	2.82	3.633 (4)	143

Symmetry codes: (i) −*x*+1, −*y*+1, −*z*+1; (ii) −*x*+1, −*y*+2, −*z*+1; (iii) *x*, *y*−1, *z*; (iv) *x*, *y*+1, *z*; (v) *x*+1/2, −*y*+1/2, *z*+1/2.
